# Managing knowledge hiding behaviors in Chinese enterprises: the mediating effects of power dynamics losses

**DOI:** 10.3389/fpsyg.2025.1600266

**Published:** 2025-11-13

**Authors:** Yi Liu, Chao Liu, Riccardo Spolaor, Shuaishuai Chen

**Affiliations:** 1 School of Innovation and Entrepreneurship, Shandong University, Qingdao, China; 2 China Classification, Qingdao, China; 3 School of Computer Science and Technology, Shandong University, Qingdao, China; 4 School of Mathematics, Shandong University, Qingdao, China

**Keywords:** perceived value of knowledge, loss of knowledge power, knowledge hiding, Chinese enterprises, decision making

## Abstract

This paper investigates the influence of power dynamics on knowledge-hiding (KH) behaviors in Chinese enterprises. Data were collected through questionnaires from 342 respondents. Structural equational modeling was performed to analyze the relationships among the perceived value of knowledge (PVK), loss of knowledge power (LOKP), and KH. The findings indicate that PVK is positively related to LOKP, and LOKP mediates the relationship between PVK and KH. However, several hypothesized relationships were not supported, highlighting the complexity of the mechanism underlying KH. The findings suggest that while power dynamics influence KH behaviors, their effects may vary across different contexts and conditions. This study enhances the current understanding of knowledge hiding by contextualizing power dynamics in the Chinese context. It offers a nuanced understanding of when and how knowledge power affects employees' willingness to share or withhold knowledge.

## 1 Introduction

Knowledge is widely recognized as a fundamental form of intellectual capital that has become an essential resource for enterprises to sustain competitive advantage in modern enterprises (Chatterjee et al., 2022; Connelly et al., 2019; Del Giudice et al., 2015; Ivory et al., 2007; Lee et al., 2013). Effective sharing, transferring, and applying of knowledge is vital for the success of an organization. At the individual level, such practices can promote employee creativity by enabling them to generate novel solutions through collective intelligence (Dong et al., 2017; Papa et al., 2018). At the team level, Men et al. (2019) and Xiong et al. (2021) suggest that smooth knowledge flow and application within organizations can promote stronger collaborative innovation and group creativity. Knowledge application enables long-term business performance and sustainability (Pan and Zhang, 2016; Semerci, 2019; Wang et al., 2019). Despite these benefits, employees as knowledge carriers frequently exhibit knowledge hiding (KH), such as deliberately withholding or concealing the requested knowledge (Connelly et al., 2012). These counterproductive behaviors stem from the perception of knowledge as a valuable personal asset, which can confer political power and bargaining advantage within organizations (Davenport and Prusak, 1998; Serenko and Bontis, 2016; Singh, 2019). This belief has been exacerbated when knowledge resources are scarce and valuable. Consequently, knowledge workers tend to withhold knowledge in order to preserve their power (Yuan et al., 2021), especially when sharing is perceived as weakening their social status.

The perceived value of knowledge (PVK) plays a decisive role in employees' willingness to engage in KH. In the eyes of employees, PVK is defined as a subjective evaluation of the knowledge possessed by assessing its utility, benefits, and strategic importance (Ford and Staples, 2006). It reflects how valuable these employees perceive their knowledge to be, to themselves and the organization. If employees view their possessed knowledge as highly valuable, they may perceive a greater loss of knowledge power (LOKP) when shared, which leads to KH (Huo et al., 2016). LOKP is often seen as the potential reduction in one's ability to influence others or loss of competitive advantage once knowledge is shared. For employees who share high-value knowledge, the consequence of LOKP may arise, and in this situation, they tend to practice KH. Although previous studies have examined the antecedents and outcomes of KH (Cerne et al., 2014; Wang et al., 2019), there remains a limited amount of empirical evidence, especially the gap lies in the explanation of how power dynamics mediates the relationships between PVK and KH behaviors, and under what cultural conditions these mechanisms operate. Indeed, the cultural context is particularly vital in the Chinese context. Arguably, in a collectivist culture like China, where hierarchical relationships and power distance significantly influence how workers interact in the workplace (Chinese Culture Connection, 1987; Jacks et al., 2012; Miles and Goo, 2013; Pearson and Entrekin, 2001; Wei et al., 2017), the practice of KH may be more evident when compared to Western settings. To address the gap, this study was undertaken to investigate KH in Chinese organizations, focusing on the mediating role of LOKP between PVK and KH.

KH is frequently encountered in enterprises. According to Connelly et al. (2012, p. 65), KH is seen as “… an intentional attempt of organizational employees to withhold or conceal knowledge” (Connelly et al., 2012, p. 65). Unlike physical assets, knowledge is held by individual employees, who have no obligation to share, and organizations do not “own” the intellectual capital. Such an asymmetry provides opportunities for employees to control and strategically withhold their knowledge for personal gain. By possessing such intellectual capital, which “… includes abilities and resources that are valuable, rare, inimitable and non-substitutable…” (Chatterjee et al., 2021, p. 104), employees can create knowledge power to preserve their professional advantage in the workplace. A deliberate concealing of knowledge often grants employees the power to protect their perceived status, influence, or indispensability within teams. An outcome tends to be shaped by concerns over resource depletion, status erosion, or interpersonal competition. In this case, one may refer to keeping the knowledge for themselves to maintain their knowledge power. According to Davenport and Prusak (1998), knowledge is viewed as a power source that employees possess, enabling them to influence decisions, maintain an advantage, or shape interactions within the organization. This argument was made as early as the seventeenth century by the English philosopher Francis Bacon (1561–1626), who emphasized that “knowledge is power” (Issac et al., 2020). It is believed that KH can be viewed as a rational, self-protective response in the environment (Evans et al., 2014), and hiding effectively can become a competitive strategy. Such an action often occurs when knowledge sharing is not rewarded or when the exchange of information can potentially lead to LOKP within organizations. In such contexts, the decision is made to hide knowledge in order to maintain autonomy, control, or job security (Duan et al., 2022; Xiong et al., 2021). Consequently, the prevalence of this phenomenon highlights the underlying power dynamics and social exchange tensions embedded within organizational knowledge systems, which warrant examination.

Employees are inclined to share or hide their knowledge if they perceive it as valuable intellectual capital, which can be used to sustain political authority and positional power (Singh, 2019). Employees, who are concerned, will protect this source (e.g., hierarchical status within the organization), and such an action inevitably leads to KH. This hidden knowledge is viewed as intellectual capital, which acts as the fuel for obtaining political mileage within and outside the enterprise, and sharing such knowledge is considered a LOKP (Ipe, 2003; Petty and Guthrie, 2000). Because these knowledge workers invested substantial time and effort to acquire such knowledge (Duan et al., 2022; Tsay et al., 2014), the psychological ownership of this knowledge is viewed as an objective status, and it is often bound by possession and proprietary rights (Pereira and Mohiya, 2021; Shen et al., 2024). When knowledge is shared, the proprietary right and its unique power will be lost (Bednall and Sanders, 2017), which triggers the KH behavior. In this case, one could argue that the potential gains or losses of power often influence employees' decisions to share or to hide knowledge.

The PVK is one of the important factors that determines knowledge workers' tendency toward KH. For instance, Ford and Staples (2006, 2010) and Huo et al. (2016) examined the connections between the PVK and KH and found that employees with higher PVK have a greater tendency toward KH. Since KH is a personal choice, and, thus, the PVK is likely to be shaped by the psychological perceptions of a knowledge worker, including their assessment of the knowledge's strategic importance and potential consequences of sharing it. Under these circumstances, knowledge workers need to decide how, when, and with whom to share knowledge within a specific context. Recognizing such dynamics, this paper explores the relationship between the PVK and KH behaviors of employees from the perspective of knowledge power. Doing so, it aims to offer insights into how PVK shapes knowledge flows within Chinese organizations.

Although KH has garnered ample research attention, the topic of KH remains in its early stages and lacks empirical evidence (Chatterjee et al., 2021; El-Kassar et al., 2022; Peng, 2013). Most of the existing research focuses on the antecedents and consequences of KH behaviors (Cerne et al., 2014; Hernaus et al., 2019; Singh, 2019; Škerlavaj et al., 2018; Wang et al., 2019), yet limited attention has been given to why (e.g., motivations), when (e.g, situational triggers), and in what context individuals hide their knowledge. For example, by analyzing the 251 matched pair data from Chinese respondents in a large manufacturing company, Pan et al. (2018) found that KH can impair knowledge creativity by creating a distrust loop among coworkers. These disruptions can compromise organizational performance, reducing employees' creativity and process capability, and affecting interaction effectiveness. Considering the extensive nature and potentially detrimental effects of KH in the workplace, especially in a collectivist culture like China, it is imperative to investigate these antecedents in greater depth. Consequently, a contextualized understanding of this phenomenon can provide targeted managerial interventions to help organizations mitigate KH and foster a more transparent and innovative organizational environment.

This research makes several contributions to the existing KH and knowledge management (KM) literature. Specifically, this study clarifies the dimensional structure of KH. It differentiates tacit KH (deceptive behaviors such as playing dumb) from explicit KH within a collective Chinese culture. Indeed, the tacit KH incorporates both playing dumb and evasive, which are considered negative behaviors with deceptive intentions (Issac et al., 2020; Miminoshvili and Cerne, 2022). These KH behaviors can result in negative outcomes such as reducing employees' creativity, discouraging knowledge sharing, and, in turn, leading to poor teamwork and organizational performance. Jiang et al. (2018) examined the repercussions of KH on Chinese employees. They found that KH negatively influences employees' thriving in enterprises, especially their interpersonal relationships with coworkers (e.g., good guanxi) and creative performance. This effect is amplified under higher levels of organizational cynicism. The explicit KH is less related to deception because a rationalized hider can present logical and official proof/reason behind their KH behaviors (Bari et al., 2020; Connelly et al., 2012). The findings of this study are crucial to understanding why, when, and to what extent employees hide knowledge, especially in the Chinese cultural context. In addition, from the knowledge power perspective, this paper explored whether the LOKP is the cause of KH behaviors. Collectively, we identify knowledge power as the mediating mechanism that explains how PVK influences KH behaviors, thereby extending the existing knowledge in the KH research. Therefore, this study provides insightful suggestions for managers to develop effective measures to reduce KH while promoting knowledge sharing in their enterprises.

## 2 Theoretical framework

The conservation of resource (COR) theory states that knowledge workers strive to acquire, retain, and protect their valuable resources, including skills, knowledge, and social capital (e.g., political positions), to avoid resource losses while seeking opportunities to gain or create new resources (Hobfoll, 1989; Hobfoll et al., 2018). Knowledge workers perceive their professional and unique knowledge, which is mastered in the workplace, as strategic assets. These can be used to generate both economic values (e.g., financial rewards) and social values (e.g., recognition and influence), ultimately enhancing their political authority or power within the enterprise (Burmeister et al., 2019; Ma and Zhang, 2021; Pereira and Mohiya, 2021). In other words, employees are interested in acquiring and conserving both personal gains and social bonds in an organizational context (Hobfoll et al., 2018). Thus, to safeguard their valuable resources, employees often choose to hide rather than share their knowledge, especially when they perceive sharing as a loss of competitive edge.

While COR theory provides a strong lens for explaining KH as a defensive behavior aimed at resource preservation, other theoretical viewpoints offer contrasting and yet limited explanations for understanding Chinese employees' KH decisions. For example, the social exchange theory (SET) argues that employees' decisions to share or hide knowledge are influenced by their reciprocal expectations. If they see mutual benefits or gaining organizational support, they tend to share knowledge (Blau, 1964). The self-determination theory (SDT) placed great importance on the role of intrinsic motivation. This theory suggests that employees who are autonomous, competent, and have a good guanxi with their coworkers are more likely to share knowledge, regardless of potential resource loss (Deci and Ryan, 2000). Furthermore, the psychological ownership theory (POT) offers another theoretical perspective. According to the POT, when employees perceive a sense of ownership over their work or resources as personal assets, they tend to hide knowledge (Pereira and Mohiya, 2021). In the Chinese context, face-saving, job stability, social capital, and resource security are highly valued by employees. To protect what they perceive as their personal and professional “ownership” of resources, they are more likely to engage in KH behaviors.

The theories of SET, SDT, and POT provide a solid foundation for the claim that KH is not solely driven by fear of resource loss. Instead, the KH behaviors can also be shaped by relational (e.g., reciprocal relationships), motivational (e.g., intrinsic motivations), and emotional factors (e.g., psychological ownership; Shen et al., 2024). This notion is particularly evident in the Chinese context. Incorporating these perspectives, founded on COR theory, enriches the theoretical framing by acknowledging competing mechanisms, such as job stability, social capital, and resource security, behind KH.

It is generally agreed that employees who share knowledge tend to thrive and succeed. To achieve this outcome, it is necessary to investigate the underlying mechanisms of KH. Doing so, this study is likely to provide additional insights to both practitioners and researchers (Peng, 2013; Scuotto et al., 2022). For example, enterprise leaders and managers can find possible solutions to mitigate the negative effects of KH. In addition, research on the antecedents of KH is in its infancy, and, hence, more studies are required. Specifically, there is a need to understand “… the factors that truly contribute to employees' decision to hide knowledge, the degree to which context plays a role…” (Scuotto et al., 2022, p. 77). How to detect KH behaviors while developing strategies to mitigate the impacts of KH and promote knowledge sharing warrants further study (Chatterjee et al., 2021; Duan et al., 2022; El-Kassar et al., 2022; Singh, 2019; Wang et al., 2019).

Consistent with the worldwide call for further exploration of the KH research, Chatterjee et al. (2021) highlighted the imperative need for additional empirical studies on KH due to measurement ambiguities. This notion is reinforced by Connelly et al. (2019), who pointed out that the dimensions of KH are yet to be confirmed and need further empirical validation. While some scholars proposed a three-dimensional structure (i.e., evasive hiding, playing dumb, and rationalized hiding), others indicated that KH can be simplified into a two-dimensional approach of active hiding and passive hiding (Connelly et al., 2012; Connelly and Zweig, 2015). More recently, scholars such as Kmieciak (2024), Ma et al. (2020), Tan et al. (2022), and Yuan et al. (2021) suggested that bullying hiding should be incorporated as a fourth dimension into the overall KH measurement. They assert that bullying hiding may have more detrimental effects on knowledge seekers and organizational knowledge exchange than other KH behaviors. Nonetheless, this additional construct requires further empirical validation to establish its robustness and relevance to the broader KH framework.

It is generally agreed that KH is a highly context-dependent behavior, and it will be affected by many contextual factors, such as cultural values (e.g., hierarchical relationships) and organizational culture. To capture the nuances of KH behaviors, it is crucial to examine the contextual influences. For instance, by interviewing 33 migrant workers from different social and cultural backgrounds, who are employed in Slovenian multinational companies and across the faculties of the University of Ljubljana, Miminoshvili and Cerne (2022) found that members of culturally minority groups exhibited what they termed “adjustable KH” to avoid their perceived workplace exclusion and advance their perceived workplace inclusion. They claimed the adjustable KH should be incorporated into future KH measurement models. While this addition may offer a more comprehensive understanding of KH, it requires further validation. It is because KH behaviors are deeply rooted in personal and organizational decision-making processes that are likely to be shaped by intrinsic motivations, organizational contexts, and national cultures. Therefore, the applicability of these KH dimensions to Chinese-based organizations remains under-researched. The contextual social dynamics (e.g., rigid hierarchy and guanxi) within Chinese organizations are complex, necessitating a more culturally sensitive framework to fully capture KH behaviors (Duan et al., 2022; Huang et al., 2021). Therefore, this study aims to empirically identify and validate the dimensions of KH in Chinese organizations, addressing the existing research gaps in models (e.g., the inconsistency measurements of KH) and offering a more nuanced understanding of KH in non-Western culture settings (Ma et al., 2020; Tan et al., 2022; Yuan et al., 2021).

### 2.1 PVK and KH

The connections between PVK and KH lie on the premise that “… knowledge has value is an ancient proposition, and arguments about the value of knowledge have been ongoing for thousands of years” (Xu and Bernard, 2011, p. 167). The value of knowledge is perceptual, and, thus, it is vital to define its business contexts (Day and Crask, 2000). For example, some researchers argue that organizations that value workers' intellectual capital (i.e., knowledge) can gain long-term sustainability (Ford and Staples, 2006). Others believe that knowledge seekers value knowledge because it can influence job performance and organizational goal achievements (Day and Crask, 2000; Socha, 1998; Zeithaml, 1988). Indeed, Gupta and Govindarajan (2000) stated that the seekers do influence knowledge-seeking behaviors. From the knowledge owners' perspective, if they are unwilling to share knowledge because of its perceived value, this knowledge value cannot be properly evaluated, and others will be unable to determine the knowledge value as high or low (Ford and Staples, 2010; Haldin-Herrgard, 2000).

Nevertheless, sharing or hiding knowledge is often determined by the types of knowledge involved. According to Nonaka and Takeuchi (1995), knowledge is classified into explicit and tacit knowledge. Explicit knowledge refers to knowledge that is accessible through consciousness and can be communicated in a formal language (Akbar and Tzokas, 2012). In contrast, tacit knowledge is more personal and deeply tied to senses, tactile experiences, movement skills, and intuitions. Such knowledge is difficult to articulate or codify; it is acquired through a personal learning process that requires a significant devotion of time and effort to gain knowledge (Duan et al., 2022). Therefore, tacit knowledge has intrinsic value and provides practical, context-specific know-how that is often more valuable to individual employees. Being highly valuable and context-dependent, employees are more likely to conceal the tacit knowledge rather than the explicit knowledge. Unlike explicit knowledge, which can be shared easily, tacit knowledge is difficult to transfer or share with others. This type of knowledge is tied to personal expertise and skills, which are deemed as critical resources by employees, and they can use to maintain their competitive advantage. To maintain their competitive advantage and protect professional status or self-interest, employees tend to practice KH in order to withhold their tacit knowledge.

Such a notion is particularly evident in the Chinese context (Qin et al., 2021). Xiao and Cooke (2018) found that protecting themselves from external harm and self-interest (e.g., avoiding job loss or work burden) were the primary motivations for Chinese employees to engage in KH behaviors. The possession of tacit knowledge requires a considerable investment of time and energy; Chinese employees will proactively set up protection mechanisms to hide that knowledge (Duan et al., 2022; Li et al., 2015). As a result, tacit KH hampers social interaction and restrains heterogeneous knowledge-sharing activities (Duan et al., 2022). Such unwillingness to share may also be caused by the LOKP (Peng, 2013). Individuals tend to restrict access to tacit knowledge that could grant them power or benefits because they perceive it as a critical intellectual capital (e.g., critical resource) within organizations (Rajan and Zingales, 1998). The possession of tacit knowledge, viewed as critical intellectual capital, elevates their power and status (Peng, 2013), and, in turn, they are more likely to hide than share knowledge with colleagues.

Despite an increasing interest in KH behaviors within enterprises, only a few current empirical studies have been conducted to investigate the relationships between PVK and KH behaviors (Huo et al., 2016). According to Ford and Staples (2006; 2010), the PVK is considered an important factor in understanding KH because knowledge value gives individuals the leverage to obtain status, power, and rewards (Gagné, 2009; Peng, 2013; Xu, 2013). Those who own valuable knowledge may have a competitive advantage over other colleagues (Gray, 2001). Arguably, the higher the PVK, the greater the possibility of KH. In addition, the PVK weakens the positive correlation between reciprocity and knowledge sharing. Xu (2013) analyses how PVK moderates the relationship between organizational incentives and knowledge-sharing tendencies. When the PVK is low, the positive correlation between organizational rewards and willingness to share knowledge will be weakened. Similarly, Huo et al. (2016) suggested that the PVK significantly and positively moderates the relationship between individual psychological ownership and territoriality. According to the COR theory (Hobfoll, 1989), when employees feel that their key resources are threatened by actual or potential losses of social values, they will make every effort to maintain, protect, and develop critical psychological resources (Bari et al., 2020). It is believed that acquiring knowledge requires investment in time, energy, and even financial resources (Duan et al., 2022). Thus, knowledge becomes a valuable form of critical intellectual capital that can be lost when shared or retained through KH behaviors. Based on the above considerations, we propose the following hypothesis:

H1: The PVK is positively related to KH.
H1a: The PVK is positively related to tacit KH.
H1b: The PVK is positively related to explicit KH.

### 2.2 The mediating role of the LOKP

Foucault's theory of power states that knowledge is the scepter that accompanies power in the power system (Foucault, 2008). Knowledge sharing not only means transferring knowledge but also undermining the power base of individuals in the enterprise (Pan and Zhang, 2016). Davenport and Prusak (1998) identify the LOKP as a critical obstacle to knowledge sharing. In particular, when individuals believe that certain knowledge constitutes a source of power or intellectual capital, they can “privatize critical resources” to maximize their interests and consolidate their political power in the enterprise (Rajan and Zingales, 1998). Additionally, knowledge workers leverage their intellectual capital, such as competencies, capacities, and capabilities, to show their value and strengthen their political power positions to influence within the enterprise. In this case, sharing knowledge is equivalent to compromising their political power, economic values, and social values (Pan and Zhang, 2016; Recherg and Syed, 2013). Based on the findings above, we propose the following hypothesis:

H2: The PVK is positively related to the LOKP.

The anxiety of losing knowledge power is the main reason for KH among many employees (Orlikowski, 1993). It is believed that sharing knowledge could reduce their status and power in the enterprise, and hence, they tend to hide knowledge to protect these losses (Husted and Michailova, 2002). These findings have been confirmed by Renzl (2008) and Huang et al. (2008) in the Austrian and Chinese contexts, respectively. A potential LOKP and knowledge sharing are negatively correlated (He and Jiang, 2014, 2016; Zhang et al., 2017). In particular, He and Jiang (2016) argued that knowledge sharing is likely to lead to the loss of human capital control. Also, the fear of losing their value would reduce employees' willingness to share knowledge (Smaliukiene et al., 2017). Hence, these factors increase the tendency toward KH behaviors by employees. Based on the above findings, we propose the following hypotheses:

H3: The perceived LOKP has a positive effect on KH

H3a: The perceived LOKP has a positive effect on tacit KH.
H3b: The perceived LOKP has a positive effect on explicit KH.

H4: The LOKP mediates the relationship between the PVK and KH.

H4a: The LOKP mediates the relationship between the PVK and tacit KH.
H4b: The LOKP mediates the relationship between the PVK and explicit KH.

**Figure 1:**
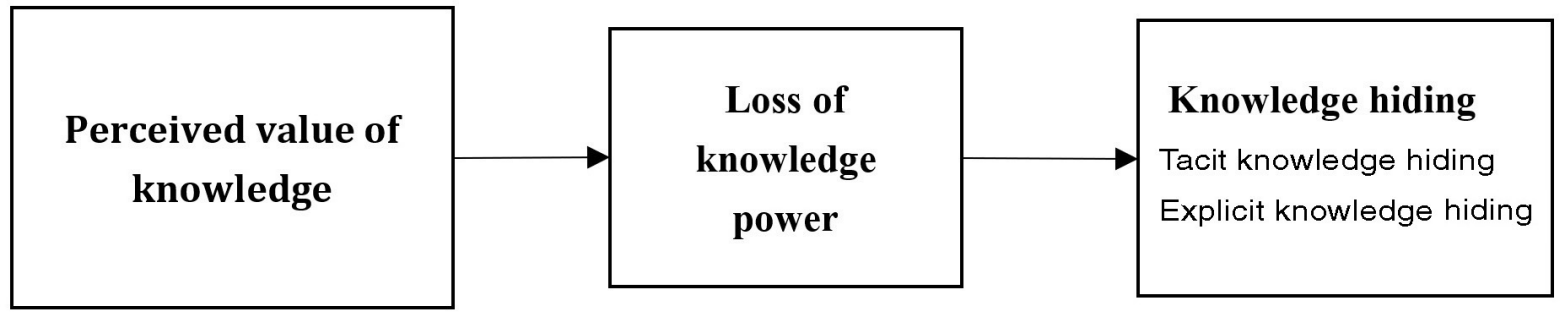
Research model.

Figure 1 presents the relationships among the investigated variables.

## 3 Research method

### 3.1 Subjects and data collection

This paper investigates employees' KH behaviors in Chinese enterprises. The final questionnaire, which was modified after a pilot study, was distributed to knowledge workers in China. This study sites were purposely selected to cover economically significant and diverse regions ranging from the nation's largest provincial economy (Guangdong with US$1.99 trillion GDP growth in 2024) to major coastal (Shandong with US$1.38 trillion GDP growth in 2024, ranking third among all provinces), central (Hubei with US $843 billion, approximately 4.45% of national GDP), and metropolitan (Shanghai with US $664 billion, contributing approximately 3.69% to the national economy) economic hubs (National Bureau of Statistics of China, 2025). Although this purposive regional coverage enhances heterogeneity and contextual richness of the study samples, we acknowledge that this distribution may limit the generalizability of the study findings. This issue was addressed in the limitations section. Nevertheless, our study provides a groundwork for future scholars to cover respondents from more diverse regions with different organizations across various sectors.

The questionnaire is mainly distributed through two channels: (1) 191 copies were sent out in the way of “snowballing” through interpersonal relationships and then retrieved, and (2) 212 copies were collected via an online survey, named wenjuanxing (https://www.wjx.cn/). A snowball sampling method, through the use of interpersonal relationships, was deemed appropriate in the Chinese context because access to organizations is often restricted. This point was reinforced by Peng (2013), who argued that the unique challenges of conducting empirical studies in China are due to its size, culture, and socio-economic complexities. To complement this data collection method, we also used an online survey, which is economical, efficient, and effective (Liu et al., 2019). The use of online surveys yielded a large response rate, enabling the researchers to produce in-depth results within a relatively short time, especially in the Chinese context (Atkinson and Flint, 2001; Heckathorn, 2011). Arguably, the combination of sampling choices collectively enhances the relevance and validity of our data, allowing us to capture the essential dynamics underpinning our theoretical model.

A total of 403 copies were received, after screening for completeness and eligibility, 342 questionnaires were identified as usable, yielding an effective response rate of 84.9%. To preserve data integrity and ensure the scientific validity of the results, outlier analysis, using Mahalanobis distance and standardized residuals (Aguinis et al., 2013), was carefully addressed before commencing statistical analyses.

### 3.2 Measures

To ensure the reliability and validity of the study, this paper measures the required variables by adapting Western scales. They have been revised to be applied in the context of Chinese enterprises. To guarantee an accurate translation of the questionnaire, a back-translation procedure was used when translating the English questionnaire into a Chinese version. To verify the accuracy of the translation process, content validity checks were undertaken by five academic experts in the fields of KM and organizational behavior, as well as five managerial practitioners from Chinese enterprises. Their feedback further validated that the retained items effectively reflected the investigated framework. Variables were measured using a seven-point Likert scale (i.e., “1”–“7” means from “strongly disagree” to “strongly agree”).

The *PVK* was measured using a nine-item scale adapted from Ford and Staples (2006). Before conducting exploratory factor analysis (EFA), the KMO and Bartlett's sphericity tests were performed. The KMO value for the PVK scale was 0.64, which indicated marginal adequacy, and the Bartlett's test of sphericity was significant. This result suggests that the data were suitable for factor analysis. The performance of EFA revealed only one factor with an eigenvalue greater than 1, accounting for 53.92% of the total variance. This finding indicates that the perceived knowledge profitability and knowledge usefulness are not empirically distinct. Indeed, follow-up interviews with some participants revealed that profitability and usefulness are inseparable aspects of knowledge value; both are ultimately tied to value creation in the studied Chinese organizations. Therefore, this study concludes that, in the Chinese context, PVK can be regarded as a unidimensional construct. The Cronbach's alpha was 0.83. A sample item includes “My work knowledge can make my work more efficient.”

*LOKP* was assessed using a four-item scale adapted from Kankanhalli et al. (2005). This scale measures individual employees' concerns about losing their exclusive rights to knowledge and their potential loss of economic, social, and power-related values within the organization upon sharing or disclosing knowledge. Furthermore, this scale tends to focus on the anxiety individuals may feel when faced with the possibility of revealing their valuable knowledge on request from colleagues. A sample item includes “When I tell my colleagues about my important work knowledge or make it public.” The Cronbach's coefficient was 0.95, indicating high internal consistency.

*KH* was measured using a scale developed by Connelly et al. (2012), which has shown high reliability and validity in the Chinese context (Cerne et al., 2014; Connelly and Zweig, 2015; Fong et al., 2018; Pan and Zhang, 2016; Zhang et al., 2017). Factor analysis revealed two subconstructs: tacit KH and explicit KH. Specifically, evasive hiding and playing dumb emerged as one construct. The merging of evasive hiding and playing dumb into a single construct was not only supported by Zhao (2013) but also aligned with existing studies in the Chinese context that treat these behaviors as deceptive strategies for concealing tacit knowledge (Duan et al., 2022; Huo et al., 2016; Zhang et al., 2017). However, we acknowledge that this merging of a single construct requires further psychometric validation in different cultural and organizational contexts. A notion is supported by Connelly et al. (2019), who indicated that employees' KH behaviors may be different across cultural contexts, due to variations in perceptions of deception and knowledge ownership. For instance, Zhang et al. (2017) argued that employees in collectivist cultures like China are more likely to adopt deceptive strategies to engage in KH to preserve their tacit knowledge and social capital. In this context, individual employees deliberately adopt these strategies to protect their tacit knowledge, such as intellectual capital, and avoid potential LOKP when faced with requests from knowledge seekers. Accordingly, we have termed this dimension as *tacit KH*, which was measured with an eight-item scale. A sample item includes: “When a colleague asks me for knowledge, I might say that I don't know much about this topic.” The *explicit KH* was measured using a four-item scale, and a sample item includes “When a colleague asks me for knowledge, I may explain that I want to tell him, but I am not allowed to do so.” The Cronbach's alphas were 0.90 and 0.76, respectively.

*Control variables* were also measured in this study. Reviewing the existing literature, it is noted that employees have different perspectives and ideological levels. They may respond differently to the knowledge requests by others and, therefore, affect KM behaviors (Connelly et al., 2012; Liu et al., 2019). The control variables include employees' background information such as gender, age, educational background, job tenure, job position, organizational type, company size, and industry.

### 3.3 Data analysis

Data collected from a single source using the same method may be affected by measurement errors because of Common Method Variance (CMV; Podsakoff et al., 2003, 2012; Richardson et al., 2009). To avoid the effects of CMV, this study collected data through two channels: personal connections and online surveys. According to Park and Lee (2014), data collected from multiple channels can potentially reduce the threats of CMV. To further yield the validity of the study findings, the examined variables were measured separately. Specifically, through personal connections, a total of 191 knowledge workers completed the survey, which measured the PVK and LOKP. The construct of KH was assessed by an online survey with 212 participants involved in this part of the study.

To detect whether CMV and Common Method Bias (CMB) influence the study findings, several methods were used. For instance, Harman (1976) single-factor test was performed on SPSS 23. The statistical results indicated the highest covariance explained by one factor was 21.96% (less than 0.5), which suggests CMV was unlikely to exist. Another method used to check whether the studied data suffer from CMB is to look at the correlations between the investigated variables. According to Bagozzi et al. (1991), a correlation value greater than 0.90 between the studied variables may indicate the presence of common method variance (CMV). As shown in Table 2, the highest correlation between any two studied constructs is 0.67, further confirming that CMB is not an issue in the present study.

Confirmatory factor analysis (CFA) was performed to test whether the scale has good discrimination. For example, CFA was conducted on the multidimensional construct of KH. The two-factor model of knowledge hiding demonstrated good model fit, with χ^2^/*df* = 3.04 (< 5), RMSEA = 0.077 (< 0.08), and CFI, TLI, and IFI values of 0.96, 0.94, and 0.96, respectively—all exceeding the 0.9 threshold. These results indicate that the two-factor model provides an acceptable fit to the data. In contrast, the single-factor model showed a poor fit, with χ^2^/*df* = 16.13 (>5), RMSEA = 0.221 (>0.08), and CFI, TLI, and IFI values all below 0.9, suggesting inadequate model fit (Table 1).

**Table 1:** Results of KH scale discriminant validity tests.

**Model**	**χ^2^**	***df***	**χ^2^/*df***	**RMSEA**	**CFI**	**TLI**	**IFI**
Two-factor model (TKH; EKH)	158.31	52	3.04	0.077	0.96	0.94	0.96
One-factor model (TKH+EKH)	871.11	54	16.13	0.211	0.66	0.58	0.66

*n* = 342, TKH, Tacit knowledge hiding; EKH, Explicit knowledge hiding.

The assessments conducted in AMOS 23 indicated the construct validity-related indicators of the total scale meet the standards, with the incremental fit index (IFI) = 0.918, comparative fit index (CFI) = 0.917, and root mean square error of approximation (RMSEA) = 0.064 (Hair et al., 2010), which suggests the model fits the data well.

## 4 Results

Participants' demographic information was reported from two perspectives. From a personal perspective, the number of male (52.8%) and female respondents (48.2%) is roughly equal. A majority of the respondents were relatively young, with two-thirds of them aged over 30 years (63.5%). 91.8% (i.e., undergraduate and above) of the knowledge workers had high educational backgrounds. An even distribution was found in respondents' job tenure, and most of them (i.e., 95.9%) were middle-level employees within the organization. From an organizational perspective, the private and state-owned enterprises occupied 41.2% and 31.9%, respectively, and the rest were government and public institutions (13.5%) and foreign enterprises (13.5%). Regarding organizational sizes, 14.9% of enterprises had less than 100 employees, 36% had between 101 and 499 employees, 11.7% had 500 to 999 employees, and 37.4% had more than 1,000 employees occupied 37.4%. The studied respondents captured knowledge workers from several industries, including manufacturing, Internet, education, and finance.

The means, standard deviations, correlations, and reliability coefficients of the investigated variables are reported in Table 2. Most of the variables are significantly related to one another. For example, the PVK is positively related to LOKP (*r* = 0.67, *p* < 0.01), tacit KH (*r* = 0.14, *p* < 0.05), and explicit KH (*r* = 0.15, *p* < 0.01). The LOKP is positively related to tacit KH (*r* = 0.17, *p* < 0.01) and explicit KH (*r* = 0.19, *p* < 0.01).

**Table 2:** Mean, standard deviation, and correlations.

**No**.	**Variables**	**M**	**SD**	**1**	**2**	**3**	**4**	**5**	**6**	**7**	**8**	**9**	**10**	**11**	**12**
1	Gender	0.48	0.50												
2	Age	2.49	1.14	−0.14^**^											
3	Education	3.23	0.60	−0.11^*^	−0.31^**^										
4	Job tenure	3.05	1.48	−0.01	0.79^**^	−0.53^**^									
5	Position	1.56	0.57	0.01	0.38^**^	−0.37^**^	0.57^**^								
6	Organizational type	2.82	1.11	0.18^**^	−0.09	−0.19^**^	−0.01	0.04							
7	Company size	2.72	1.12	−0.24^**^	−0.15^**^	0.36^**^	−0.27^**^	−0.22^**^	−0.05						
8	Industry	3.61	2.96	0.09	0.06	−0.14^**^	0.06	0.04	−0.01	−0.30^**^					
9	PVK	5.27	0.60	−0.10	0.06	−0.04	0.06	0.20^**^	0.05	0.05	0.02	**(0.89)**			
10	LOKP	5.21	0.62	−0.09	0.02	0.02	0.04	0.08	0.01	0.08	−0.11	0.67^**^	**(0.91)**		
11	Tacit KH	3.04	1.22	0.09	0.11^*^	−0.14^*^	0.22^**^	0.18^**^	−0.09	−0.20^**^	0.10	0.14^*^	0.17^**^	**(0.90)**	
12	Explicit KH	4.19	1.62	0.13^*^	0.10	−0.15^**^	0.19^**^	0.20^**^	−0.03	−0.14^**^	0.08	0.15^**^	0.19^**^	0.38^**^	**(0.82)**

M, Mean; SD, standard deviation of mean; the numbers in brackets are Cronbach α coefficients; *n* = 342; ^*^p < 0.05, ^**^p < 0.01, PVK, Perceived value of knowledge; LOKP, Loss of knowledge power; KH, Knowledge hiding. Bold values in brackets are reliability scores.

Interestingly, most of the control variables, except gender, organizational type, and industry, were related to the studied variables. While these significant findings might be a concern, previous studies have found that age, education, job position, and company size as controls have also been found to be related to several study variables (Connelly et al., 2012; Durst and Wihelm, 2012; Lin, 2011; Liu et al., 2019; Magnier-Watanabe and Sonoo, 2008; Peng, 2013). Specifically, age is positively related to tacit KH (*r* = 0.11, *p* < 0.05), but not explicit KH. Education is significantly related to tacit KH (*r* = −0.14, *p* < 0.05) and explicit KH (*r* = −0.15, *p* < 0.01). Job position is positively related to PVK (*r* = 0.20, *p* < 0.01), tacit KH (*r* = 0.18, *p* < 0.01), and explicit KH (*r* = 0.20, *p* < 0.01). Company size is significantly related to tacit KH (*r* = −0.20, *p* < 0.01) and explicit KH (*r* = −0.14, *p* < 0.01).

**Figure 2:**
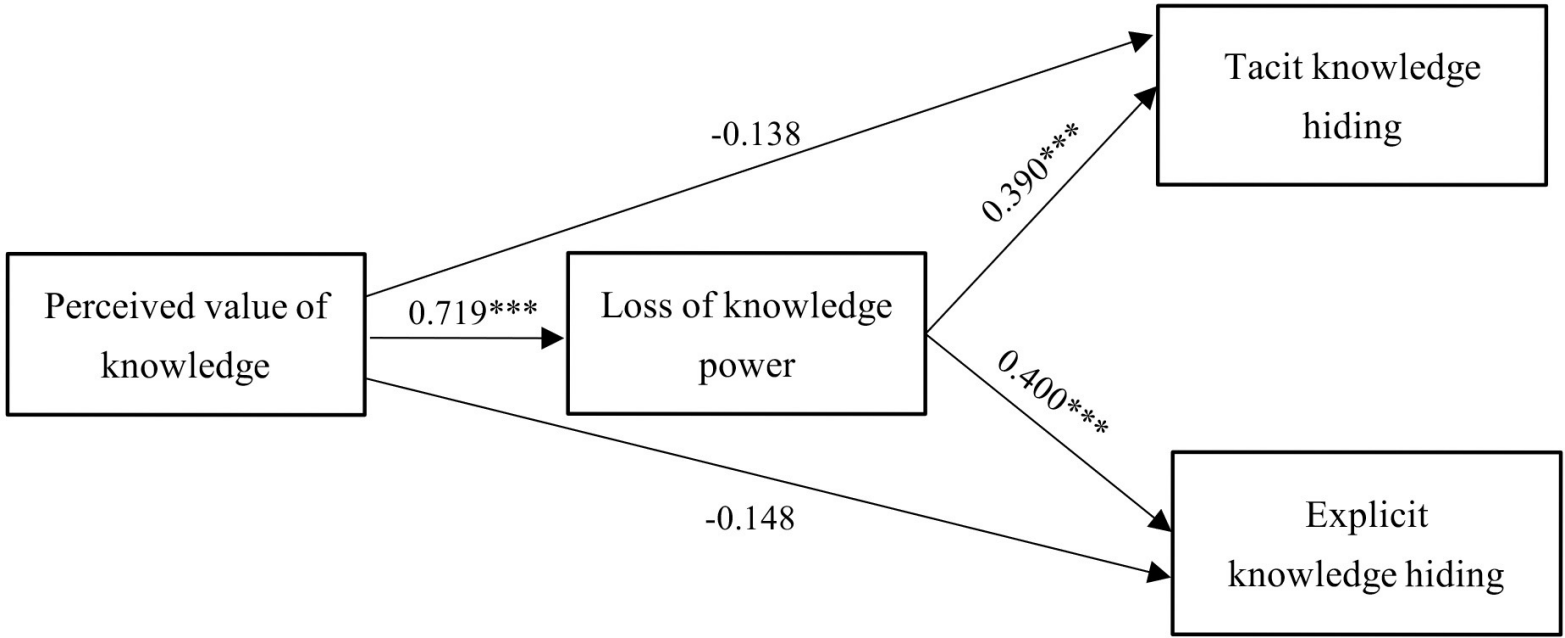
Results of path analysis. ***p < 0.001.

Figure 2 presents the results of the path analysis. The PVK is not related to KH, namely the tacit KH (H1a) and the explicit KH (H1b), respectively. Thus, hypotheses H1, H1a, and H1b are not supported. In addition, the PVK is positively related to the LOKP (β = 0.719, p < 0.001); hence, H2 is supported. Furthermore, the LOKP is positively related to the tacit KH (β = 0.390, p < 0.001, H3a) and the explicit KH (β = 0.400, *p* < 0.001, H3b). Hence, H3, H3a, and H3b are supported.

Table 3 summarizes the mediating effects. It is shown in Table 3 that the LOKP positively mediates the relationship between the PVK and KH. The indirect effect of PVK on tacit KH through LOKP is significant (β = 0.198, standard error (S.E.) = 0.102, 95% Confidence Interval (CI): [0.008, 0.414]). The confidence interval did not contain zero, thus confirming the presence of a significant indirect effect. Such a finding provides support for the claim that LOKP significantly mediates the relationship between PVK and tacit KH. Similarly, the indirect effect of PVK on explicit KH through LOKP is also significant (β = 0.293, S.E. = 0.136, CI: [0.036, 0.571]). The results reveal that zero was excluded in the confidence interval, providing strong evidence for the mediating role of LOKP in the relationship between PVK and explicit KH. These bootstrapping results strongly support H4, H4a, and H4b, indicating that LOKP plays a significant role in mediating the effects of PVK on both tacit KH and explicit KH hiding behaviors.

**Table 3:** The mediating effect of LOKP.

**Path**	**Estimate**	**S.E**.	**CI**
PKV-LOKP-TKH	0.198^**^	0.102	0.008, 0.414
PKV-LOKP-EKH	0.293^**^	0.136	0.036, 0.571

^**^p < 0.01, *n* = 342, PKV, Perceived knowledge value; LOKP, Loss of knowledge power; TKH, Tacit knowledge hiding; EKH, Explicit knowledge hiding.

## 5 Discussion

This study investigates how PVK influences employees' KH behaviors, including tacit KH and explicit KH within Chinese enterprises, and explores the mediating role of LOKP in this relationship. Drawn on the COR theory (Hobfoll, 1989), the study findings offer some valuable insights for enterprises seeking to understand how employees' perceptions of knowledge value influence their willingness to share or hide knowledge. Specifically, this research suggests that LOKP mediates the relationship between the PVK and KH behaviors in the studied Chinese enterprises. When employees perceive knowledge as valuable, granting them power, social status, and economic benefits, they are more likely to hide knowledge to avoid potential losses. This finding aligns with previous research stating that KH is seen as a strategy that employees tend to use to sustain their knowledge power (Hernaus et al., 2019; Issac et al., 2023).

While acknowledging the direct effect between PVK and KH (H1) was not supported, mediation can still be tested under the theoretical framework of indirect-only mediation paths. Supported by Zhao et al. (2010), the indirect mediation can be justified even in the absence of a significant direct effect if there is a strong theoretical rationale for the mediation process. The COR theory used in our study provides a strong foundation for our attempts to investigate the important role LOKP plays in mediating the relationship between PVK and KH. According to the COR theory, if there is an increase in the psychological cost of sharing a knowledge resource that is perceived as valuable, employees will choose to hide knowledge to prevent potential losses in power and status. Thus, the indirect path through LOKP can be theoretically justified, even though the direct relationship between PVK and KH was not significant.

The findings of this study are consistent with mainstream views, which state that a high PVK leads to the following four effects. First, a higher PVK often results in the desire to hide knowledge in order to attain benefits, such as gaining the attention of superiors and colleagues, and maintaining job security, and, thus, it is worthy of being concealed (Duan et al., 2022; Hobfoll, 1989; Rajan and Zingales, 1998; Xiong et al., 2021). Second, employees perceive that knowledge is power because it helps improve work-related outcomes such as efficiency, overcoming challenges, and achieving goals (Cerne et al., 2014; Duan et al., 2022; Ma et al., 2020; Pacharapha and Ractham, 2012). Third, the uniqueness of knowledge further motivates employees to maintain a distinct competitive advantage within their enterprise, while sharing knowledge means weakening its uniqueness (Pan and Zhang, 2016). Fourth, the significant resource required to acquire high-value knowledge (i.e., time, energy, and resources), which makes them perceive knowledge sharing as potentially unfair (Davenport and Prusak, 1998; Huo et al., 2016; Issac et al., 2023). In other words, the higher employees perceive their knowledge as valuable, the higher the psychological cost of sharing that knowledge (Xu, 2013). Consequently, those employees often hide their knowledge to avoid possible LOKP. Collectively, we believe our study findings offer new insights into how knowledge value perceptions shape employees' behavior toward knowledge sharing and hiding in the Chinese context in general, in the studied organizations in particular.

Figure 2 shows that the regression coefficient between PVK and LOKP is 0.719 (*p* < 0.001), and the regression coefficients between LOKP and the explicit KH are 0.390 (*p* < 0.05) and 0.400 (*p* < 0.05), respectively. It can be observed that the greater PVK held by an individual, the higher the LOKP I associated with sharing knowledge, resulting in a stronger tendency to practice KH. In the era of the knowledge economy, employees who retain intellectual capital, such as critical knowledge and resources, have a competitive knowledge advantage (Chatterjee et al., 2022; Connelly et al., 2019; Del Giudice et al., 2015). In other words, “knowledge represents the power to manage and control everything, and the implicit and complex knowledge advantages of individuals cause them to become a strong force in knowledge exchange, which will form a consciousness and concept of ‘knowledge power' based on knowledge advantages” (Yuan et al., 2021, p. 1370). For knowledge workers, high-value knowledge can attain political power, gain the respect and assertiveness of their bosses and colleagues, maintain high work efficiency as well as unique economic and social values (Bari et al., 2020; Burmeister et al., 2019; Davenport and Prusak, 1998; Duan et al., 2022; Ma and Zhang, 2021). Sharing such high-value knowledge means handing out this unique power to others, which is counterproductive in a highly competitive organizational environment (Connelly and Zweig, 2015; Pan and Zhang, 2016; Singh, 2019); hence, employees are more likely to engage in KH behaviors.

According to the COR theory, when employees perceive a loss of their essential intellectual capital, such as critical resources or a sense of potential threats, they would maximize their efforts to maintain and protect such resources, thereby avoiding the loss of information resources (Hobfoll, 1989). Ultimately, these study findings align with previous research indicating that Chinese knowledge workers are more likely to hide knowledge as a means of retaining knowledge power (Davenport and Prusak, 1998; Orlikowski, 1993; Zhang et al., 2017). For knowledge workers, high-value knowledge represents their value, status, and a source of power base. Sharing or publicizing high-value knowledge is equivalent to losing their power base in the enterprise (Elaimi and Persaud, 2014; Recherg and Syed, 2013), which can compromise their irreplaceability in the enterprise. Therefore, employees are most likely to hide knowledge to protect their interests and position.

## 6 Research and practical implications

This paper contributes to the COR theory by investigating the mediating effect of knowledge power in the relationship between the PVK and KH. Our study extends COR theory by highlighting how employees' perceptions of knowledge power, as a valuable resource, influence their knowledge sharing and hiding behaviors. Specifically, we demonstrate that the LOKP serves as a direct factor leading to employees' KH behaviors. Indeed, when employees perceive a loss of power, status, or competitive advantage as key resources within the COR framework for sharing knowledge, they are more likely to engage in KH. Surprisingly, as an unexpected result, the lack of a direct relationship between PVK and KH contradicts our initial hypothesis. Such a finding suggests that the relationship is more complex and requires a more nuanced understanding within a specific context. Indeed, the relationship between PVK and KH is mediated through factors, such as the perceived LOKP and other associated risks. This unexpected result provides rich avenues for future scholars to investigate why PVK does not have a direct impact on KH. A possible explanation is that PVK alone is insufficient to drive employees to engage in KH, unless there are perceived risks (e.g., LOKP) associated with sharing knowledge. This valuable insight contributes to refining the application of COR theory to be context-specific organizational knowledge dynamics. Collectively, in line with COR theory, these study findings deepen the understanding of how resource loss (e.g., LOKP) triggers employees' defensive behaviors to engage in KH.

In the Chinese managerial context, organizations need to make KH a strategic issue. Specifically, to mitigate or avoid KH behaviors, it is vital to address the psychological and material costs associated with knowledge sharing. Data collected from 190 knowledge workers in China reveal that organizations should focus on practices that can decrease employees' perceived knowledge possession and knowledge territoriality, and such an action can reduce the chances of engaging in KH (Peng, 2013). In this case, Chinese organizations need to improve their KM incentives by creating a secure and equitable compensation system to alleviate employees' perceived LOKP. More specifically, rewards should be given to employees who share knowledge to reduce employees' sense of knowledge power loss (Wen and Ma, 2021). By studying 466 managerial employees in Chinese financial institutions, Wen and Ma (2021) stated that internal competition and a lack of reward for knowledge sharing often drive employees to engage in KH. Collectively, an equitable and transparent KM system includes intangible rewards such as formal recognition, financial rewards, and career ladder opportunities tied to collaborative behaviors among employees can not only reduce internal competition but also foster knowledge sharing.

These initiatives are particularly important in the collective Chinese context, where hierarchical relationships and power distance, trust building significantly influence the interaction among employees at work (Chinese Culture Connection, 1987; Jacks et al., 2012; Liu et al., 2019; Miles and Goo, 2013; Pearson and Entrekin, 2001; Wei et al., 2017). Such efforts not only reduce KH behaviors but also enhance the overall organizational knowledge flow and collaboration. These findings extend COR theory by linking resource loss to KH. More importantly, we suggest that organizations need to manage knowledge by improving KM practices to reduce employees' perceived risks associated with sharing valuable knowledge. Thus, understanding employees' knowledge power within an organization is crucial for grasping the nuances of their KH behaviors.

This study also revealed that Western-developed and tested instruments have good psychometrics in the Chinese context. Specifically, the validity and reliability of the measures for the studied variables demonstrate good psychometric properties and lay the foundation for researchers to employ them in future studies in China. However, this study has adapted the instruments for the PVK and KH to contextualize the statements. The adapted 12-item scale was kept to measure the construct of KH, and the results of factor analyses led to interesting findings. For example, the initial KH scale, developed by Connelly et al. (2012), had three sub-constructs: evasive hiding, playing dumb, and rational hiding. However, Chinese participants viewed evasive hiding and playing dumb as forms of deception, which involves deliberate procrastination of individuals in the face of knowledge requests from others. This cultural interpretation led to an overlap of these unique characteristics in evasive hiding and playing dumb (the tacit KH), further confirming the importance of cultural context when undertaking cross-cultural studies.

Similarly, this distinctive feature was also found in the measurement of the PVK. The initial four factors of benefits (four items), usefulness (five items), uniqueness (three items), and source (three items) were adapted from Ford and Staples (2006) to measure the construct of the PVK. However, this study did not fully capture the knowledge value construct in the Chinese context. We found that benefits and usefulness were the main features contributing to employees' PVK. The sub-dimensions of uniqueness and source were less relevant to the measurement of PVK due to low-reliability scores. Hence, the decision was made to remove them from the PVK scale. This surprising finding can be explained from a cultural perspective. In Chinese organizations, the emphasis tends to be placed on the practical and utilitarian aspects of knowledge (i.e., benefits and usefulness), rather than its uniqueness or origin. A finding was supported by Liu et al. (2019), who indicated that Chinese organizations nowadays focus on converting knowledge into practice. It is called xue yi zhi yong (学以致用) in Chinese. These organizations encourage their employees to possess useful knowledge that helps promote holistic thinking and be innovative. Hence, they focus on internalizing knowledge that is beneficial for solving problems and improving efficiency in their workplaces. Thus, the dimensional reduction of the PVK scale further confirms the belief that adapting and applying theoretical constructs in cross-cultural studies should be context-specific and culturally sensitive.

This study also contributes to previous findings on knowledge sharing culture within Chinese organizations, emphasizing the importance of continuous learning (Gourlay, 2004; Liu et al., 2019). Continuous learning organizations place a great importance on creating a knowledge culture that enables knowledge flows among members. This kind of organizations has exceptional advantages in encouraging team learning (Prusak, 2016). As a more effective environment for learning, a knowledge-sharing culture promotes harmonized relationships among members and accelerates knowledge exchange. Learning organizations require employees to make continuous progress, to engage in “best practices” (Caniëls et al., 2017), be innovative, and advocate for continuous learning and sustainable development. In other words, it is crucial to create a learning culture, as some Chinese organizations have already done so (Liu et al., 2019; Song, 2015), to reduce the occurrence of KH behaviors. Indeed, organizations that advocate knowledge sharing can potentially reduce KH behaviors among employees. To achieve such an outcome, Chinese organizations need to establish a knowledge-sharing culture that prioritizes continuous learning, encourages knowledge sharing, and knowledge acquisition among employees. By doing so, in a collective cultural context like China, these organizations are more likely to realize the importance of knowledge sharing for personal improvement and sustainable development (Latif et al., 2020; Mura et al., 2013; Pradhan et al., 2020; Ramadan et al., 2017). As a result, employees are more likely to share knowledge to mitigate the possibility of KH (Cerne et al., 2014; Ford and Staples, 2010).

## 7 Limitations and future research

While this study provides valuable insights, the findings should be interpreted with the following limitations. First, the scales used to measure study variables, such as KH and PVK, were tested with samples from Western contexts, and their applicability with samples from the Chinese context requires further validation. According to Liu et al. (2019), the political backgrounds, cultural differences, and organizational settings can potentially affect how KH behaviors are interpreted. These differences can alter the study findings.

Second, the study samples are collected from certain geographical regions using the snowball sampling method. Such an action may limit the generalizability of the study findings. The survey participants are concentrated in provincial-level cities, such as Shandong Qingdao and Hubei Wuhan. These restrictions could potentially influence the generalizability of the study findings. Additionally, the data were collected from all levels of employees without distinguishing their perceptual differences. Yet these individuals tend to hold different perceptions of KH behaviors. The differences may cause variability in the process of decision-making to act or not to act on KH.

Third, authors used self-reported questionnaires, which are essentially cross-sectional data. Although a cross-sectional design provides a valuable snapshot of the relationships among variables, it inherently limits the possibility of drawing causal inferences or capturing dynamic interactions as they progress. Specifically, the temporal sequence of how PVK influences LOKP and how these, in turn, affect employees' knowledge hiding intentions should be further evaluated.

To further validate the study findings, scholars in the future may want to examine employees' intentions to engage in knowledge hiding behaviors through a longitudinal design. Such a design can potentially capture the psychological behaviors over time, because the value of knowledge progresses with time and the accumulation of working experiences. Another worthwhile pursuit approach that future research may adopt is the employment of mixed methods. An integration of both qualitative insights (e.g., case studies or interviews) and quantitative samples could help researchers gain a deeper appreciation of contextual mechanisms. Additionally, scholars may wish to expand sample coverage by incorporating different regions and enterprises to enhance external validity, while allowing for comparative studies with more sophisticated statistical analyses (e.g., SEM). Moreover, future researchers can also partition the data according to company sizes (e.g., small and medium vs. large enterprises) or sector types to comprehensively test whether the observed effects are generalizable or context-specific. Collectively addressing these limitations can provide a more robust and nuanced understanding of the mechanisms underlying knowledge hiding behaviors in general, and in the Chinese context in particular.

## 8 Conclusion

Knowledge is a primary source of competitive advantage, fostering a knowledge-sharing culture that promotes highly coordinated actions is widely recognized as a key to sustaining competitive advantage and driving organizational success. However, knowledge hiding remains a critical challenge for knowledge-based enterprises and a major concern in the field of KM. The study findings highlight that employees' KH is primarily driven by their LOKP. These employees often evaluate both potential losses, such as diminished power, political positions, and social status, as well as potential gains (e.g., financial rewards, recognitions, or career advancement) when deciding whether to hide or share knowledge. When the perceived knowledge losses exceeded the potential benefits, they are more likely to engage in KH behaviors. Conversely, when the benefits outweigh the losses, knowledge sharing is encouraged. Therefore, the fundamental way to reduce KH behaviors in the enterprise is to increase employees' perception of personal benefits through knowledge sharing and reduce the sense of loss.

Considering a wider perspective, KH is not inherently detrimental. When the knowledge request involves organizational secrets, intellectual property rights, or third-party interests, selective withholding may serve to protect organizational interests. Therefore, instead of focusing solely on suppressing KH behaviors, enterprises should manage them strategically by asking employees to weigh potential losses (e.g., LOKP) against potential gains in order to reduce negative forms of tacit KH based on organizational objectives and task characteristics. This nuanced understanding not only aligns knowledge management practices with enterprises' strategic objectives but also underscores the global relevance of managing KH in a way that balances organizational protection with knowledge sharing to foster sustainable competitive advantage.

## Data Availability

The datasets generated for this study are not publicly available and are only available with the permission of the surveyed organizations. Requests to access the datasets should be directed to the corresponding author.
